# Enhanced peroxymonosulfate activation for tetracycline removal using magnesium–aluminum layered double hydroxide/zeolitic imidazolate framework-8 nanocomposite

**DOI:** 10.1038/s41598-026-49645-2

**Published:** 2026-04-25

**Authors:** Zohreh Jalaledin, Ali Akbar Amooey, Shahram Ghasemi

**Affiliations:** 1 Department of Chemical Engineering, Faculty of Engineering and Technology, University of Mazandaran, Babolsar, Iran; 2 Faculty of Chemistry, University of Mazandaran, Babolsar, Iran

**Keywords:** Layered double hydroxide, Nanocomposite, Tetracycline, Peroxymonosulfate, Zeolitic imidazolate framework-8, Chemistry, Environmental sciences, Materials science

## Abstract

Advanced oxidation process (AOP) utilizing peroxymonosulfate (PMS) has emerged as effective methods for environmental remediation due to their high reactivity and selectivity. However, designing hierarchical catalysts with multiple active sites for PMS activation remains an important challenge. Here, a nanocomposite was synthesized based on zeolitic imidazolate framework-8 (ZIF-8) formed on magnesium-aluminum layered double hydroxides (Mg-Al LDH) to facilitate tetracycline (TC) removal through PMS activation. The effects of key operational parameters, including PMS dose, nanocomposite concentration, initial pH, pollutant concentration, and reaction duration, were investigated. The results showed that approximately 97.5% of TC was removed within 12 min at pH = 7, for TC concentration of 25 mg/L, Mg-Al LDH/ZIF-8 dose of 9 mg/L, PMS dose of 0.28 mM. The LDH/ZIF-8/PMS system exhibited superior efficiency for TC removal compared to Mg-Al LDH/ZIF-8, Mg-Al LDH, and ZIF-8. The process was analyzed using Langmuir, Freundlich, and Temkin isotherm models, with the Langmuir model exhibiting the highest correlation coefficient (R^2^= 0.9923), confirming a monolayer catalytic mechanism on a homogeneous surface. Results from quenching experiments indicated that sulfate and hydroxyl radicals (SO₄•⁻ and •OH) are primary species driving the degradation of TC. These findings provide valuable insights on potential application of Mg-Al LDH/ZIF-8 for environmental purification.

## Introduction

Environmental problems caused by antibiotics have been widely concerned in recent years^1^. Tetracycline (TC), a representative member of the tetracycline class of antibiotics, has been widely used in both medical treatments and animal husbandry. However, complete elimination of TC and its metabolites through natural degradation remains challenging due to the presence of antibiotic resistance genes^2^. It was reported that about 70–90% of TC could may not be metabolized by organisms, lead great environmental pollution problems^3^. Across multiple countries, the peak concentrations of TC found in surface waters have reached microgram-per-liter levels, presenting considerable threats to environmental ecosystems and public health^4,5^. To address these challenges, various established water treatment techniques have been employed to mitigate pollution in aquatic environments, such as adsorption^6^, biodegradation^7^, membrane filtration^8^, and chemical oxidation^9^. Among these, advanced oxidation processes (AOPs) demonstrate exceptional promise for TC elimination, owing to their ability to generate reactive oxygen species (ROS) in situ, which efficiently degrade TC compounds^10^.

Due to their exceptional oxidative power and eco-friendly nature, AOPs are widely applied to eliminate stubborn organic pollutants. Among these, persulfate (PS)-based AOPs have recently gained considerable momentum as promising alternatives^4,11^. Peroxymonosulfate (PMS) exhibits greater ease of activation compared to peroxydisulfate (PDS), primarily due to its asymmetric molecular configuration and shorter O–O bond length, which enhance the formation of reactive species^12^. PMS-based AOPs are regarded as efficient approaches for removal of persistent organic pollutants, largely due to advantageous properties of the sulfate radical (SO₄•⁻) produced during activation. These properties include oxidation potential of 2.5–3.1 V, a relatively extended half-life (t₁/₂ = 30–40 µs), and large pH range of 2.0–8.0^13^. The generation of SO₄•⁻ can be achieved by activating PMS through various techniques, including thermal treatment, chemical agents, ultraviolet irradiation, transition metal catalysis, and ultrasonic energy^14,15^.

Research has shown that bimetallic systems can possess superior intrinsic catalytic characteristics, thereby improving both the stoichiometric and utilization efficiency of PMS compared to monometallic counterparts^16,17^. To date, recent researches have been focused on investigation of various heterogeneous bimetallic catalysts for the activation of PMS^18,19^. Among them, layered double hydroxides (LDHs) have demonstrated outstanding efficacy in activating PMS for contaminant degradation. This performance is primarily attributed to their ion-exchange capabilities, the presence of diverse metal–oxygen functional groups, and a self-repairing layered architecture that supports their reusability^20^. LDHs are anionic double metal hydroxides with the common formula [M_1−x_^+2^ M_x_^+3^ (OH)_2_] ^+x^ (A^− n^) _x/n_ mH_2_O^21,22^. The composition and ratio of metal elements within the layers, as well as the type of interlayer anions can affect the morphologies of LDHs^23^. In other hand, MOFs are composed of a set of metal ions and organic ligands, which are excellent as substrate for catalytic purposes including photocatalysis, adsorption and chemical catalysis^24–27^. Some properties of MOFs including high porosity and surface area, tunable nodes and connections, and chemical stability are important structural features for fabrication of well-dispersed catalysts^28,29^.

Guo et al. investigated the efficient removal of sulfamethoxazole using a Cu-Co LDH composite membrane and LDH/fibers, which activate PMS^30^. Zuo et al. explored the degradation of TC by activating PMS with a novel boron-doped Ni-Co LDH catalyst^31^. Wang et al. developed polyoxometalate intercalated La-doped Ni-Fe LDH for elimination of TC via PMS activation^32^. Yang et al. investigated the activation of PMS by 2D-3D De@ Ni-Fe LDH for efficient removal of TC^33^. Mi et al. synthesized Fe-Ni LDH@ biochar composite for activation of PMS towards enhanced removal of doxycycline^34^.

In this study, magnesium–aluminum layered double hydroxides (Mg–Al LDHs) derived from ZIF-8 in the presence of PMS was proposed for removal of TC from aqueous solutions. To investigate and optimize the critical parameters namely pH, nanocomposite dosage, PMS concentration, TC concentration, and reaction time, the Central Composite Design (CCD) approach, within the framework of Response Surface Methodology (RSM), was applied. Additionally, equilibrium isotherms and kinetic models were examined to further elucidate the adsorption behavior. Mg–Al LDH/ZIF-8 nanocomposite, when activated by PMS, exhibited high efficiency in removing TC from aqueous solutions and maintained its performance over multiple reuse cycles with minimal degradation.

## Experimental section

### Materials

Al(NO_3_)_2_.9H_2_O (98%), Zn(NO_3_)_2_.6H_2_O (98%), Mg(NO_3_)_2_.6H_2_O (99%), NaOH (99%), Urea (CH_4_N_2_O), potassium peroxymonosulfate (H_3_K_5_O_18_S_4_), 2-methyl imidazole (2-MIM, C_4_H_6_N_2_, 99%), HCl (37%), CH_3_OH (98%) and C_2_H_5_OH (98%) and tetracycline hydrochloride (95%) were received from Merck.

### Characterization

X-ray diffraction (XRD) patterns were recorded using Cu K_α_ radiation (X’Pert Pro, Panalytical, Netherlands). Field emission scanning electron microscopy (FESEM, Sigma 300-HV, Germany) equipped with X-ray energy dispersive spectroscopy (EDS) was used to study the morphology and determine the elemental percentage of the samples. Fourier Transform Infrared (FT-IR) spectroscopy (Thermo Scientific, AVATAR, Germany) was performed to obtain structural information. Nitrogen adsorption/desorption isotherm was conducted on Belsorp mini II, Microtrac Bel Corp, Japan. Thermogravimetric analysis was conducted to study thermal stability of sample using TGA, Q600 (TA Instruments, USA).

### Synthesis of Mg-Al LDH/ZIF-8

**Scheme 1:**
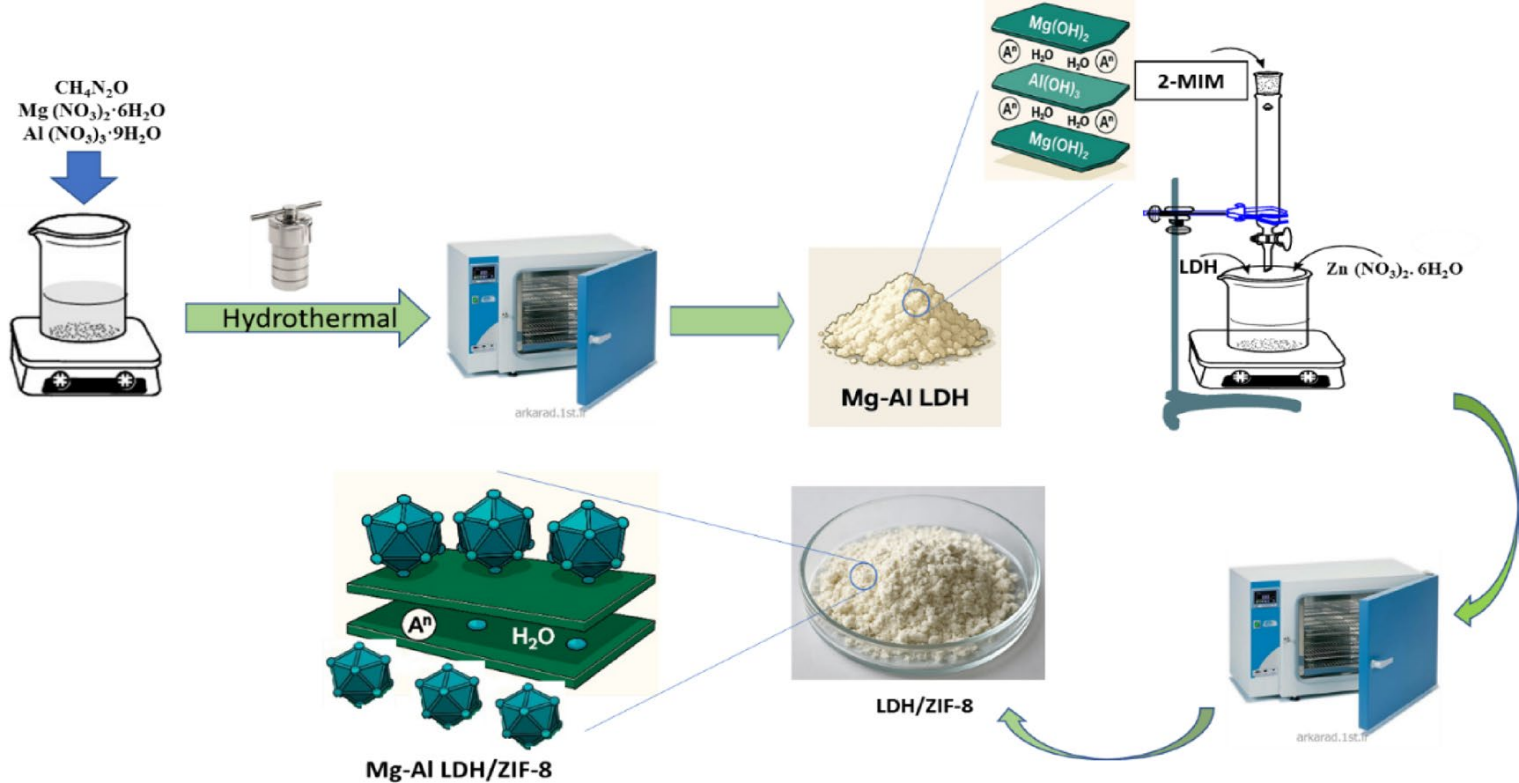
Different steps for preparation of Mg-Al LDH/ZIF-8 nanocomposite.

For preparation of Mg-Al LDH, 1 g of Mg (NO_3_)_2_·6H_2_O, 1.085 g of Al (NO_3_)_3_·9H_2_O), and 1.58 g of urea were dissolved in 40 mL methanol and stirred for 30 min and then transferred to an autoclave and heated at 150 °C for 6 h. Afterward, it was rinsed with deionized water and ethanol. Finally, the resulting white powder was dried at 80 °C for 6 h^35^. Mg-Al LDH (0.12 g) was added to 25 mL of methanol and irradiated with ultrasonic wave for 30 min. With addition of 0.446 g of Zn (NO_3_)_2_, the mixture was stirred for another 1 h (referred to as solution A). Separately, 0.492 g of 2-MIM was dissolved in 25 mL of methanol for 20 min (referred to as solution B). Solution B was slowly added dropwise to solution A under continuous stirring, and the resulting mixture was maintained under agitation for 24 h. The final product was washed thoroughly with ethanol and subsequently dried at 70 °C^36^. Scheme 1 shows different processes for the synthesis of Mg-Al LDH/ZIF-8.

### Central composite design

Design-Expert v11 software was utilized to apply RSM and CCD, enabling the assessment of key variables, their interactions, and quadratic effects. This approach facilitated experimental efficiency by minimizing the number of required runs and optimizing resource utilization. Table 1 presents the investigated factors affecting removal efficiency as response variable. A total of 32 experimental runs were performed based on CCD methodology. In Table 2, the specific values and conditions of the tested parameters are shown, which defines the experimental matrix used to optimize the four key components.

**Table 1:** Influential factors and their scope.

Affecting factors	-α	−1	0	+ 1	+α
A: TC concentration (mg/L)	10	22.5	35	47.5	60
B: Contact time (min)	5	7.5	10	12.5	15
C: Catalyst dosage (mg)	3	6	9	12	15
D: PMS dosage (mM)	0.04	0.13	0.22	0.31	0.4
E: pH	4	5.5	7	8.5	10

**Table 2:** Experiments designed with CDD to remove TC.

Experiment	Influential factors					Response	
	Concentration (mg/L)	Contact time (min)	Catalyst dosage (mg)	PMS dosage (mM)	pH	Removal efficiency (%)	
						Experimental	Predicted
1	35	10	9	0.22	7	77	80.52
2	23	8	12	0.13	6	65	65.77
3	48	13	12	0.13	6	56	57.92
4	35	10	9	0.04	7	57	58.14
5	35	10	9	0.4	7	75	75.86
6	35	10	9	0.22	10	73	73.43
7	23	8	6	0.13	9	62.5	62.92
8	748	13	6	0.13	9	68	68.20
9	35	10	9	0.22	7	81	80.52
10	23	8	6	0.31	6	60.5	61.06
11	23	8	12	0.31	9	82.7	85.34
12	35	5	9	0.22	7	67	68.6
13	35	10	9	0.22	4	63	64.58
14	23	13	12	0.31	6	96	99.18
15	10	10	9	0.22	7	97	93.73
16	48	13	12	0.31	9	80	77.5
17	48	8	12	0.31	6	70	67.48
18	60	10	9	0.22	7	63	67.31
19	23	13	6	0.13	6	56	64.08
20	35	15	9	0.22	7	90	92.45
21	48	13	6	0.31	6	70	66.34
22	48	8	6	0.13	6	50	45.51
23	35	10	3	0.22	7	39	42.66
24	35	10	9	0.22	7	83	80.52
25	35	10	9	0.22	7	83.7	80.52
26	35	10	9	0.22	7	85	80.52
27	48	8	12	0.13	9	56.8	56.76
28	48	8	6	0.31	9	53	52.5
29	35	10	15	0.22	7	71	69.34
30	23	13	12	0.13	9	90.6	88.46
31	23	13	6	0.31	9	72	71.07
32	35	10	9	0.22	7	76.5	80.52

### Degradation experiments

At first, the calibration curve for TC (y = 0.0318x + 0.0252) was obtained at a wavelength of 357 nm using a UV-Vis spectrophotometer (UV-4800 UNICO). To test catalytic performance of catalyst, batch experiment was conducted in a 150 mL conical flask. To a 50 mL of TC solution with defined concentration, the LDH/ZIF-8 catalyst and PMS were added and stirred with magnetic stirrer at room temperature. After a specified duration, 2 mL of solution were taken and filtered using a 0.22 μm membrane, and TC concentration was determined. To adjust the pH of antibiotic solution, 0.1 M NaOH or HCl was used. The equation related to TC elimination is defined as following equation:

1 $$\:R\left(\%\right)=\left(\frac{{C}_{0}-{C}_{t}}{{C}_{0}}\right)*100\:$$

C_t_ (mg/L) and C_0_ (mg/L) are TC concentrations at time t and t=0. The investigation primarily addressed the reaction kinetics, degradation mechanisms, and system performance under optimized operational conditions.

## Results and discussion

### Characterization of catalyst

**Fig. 1:**
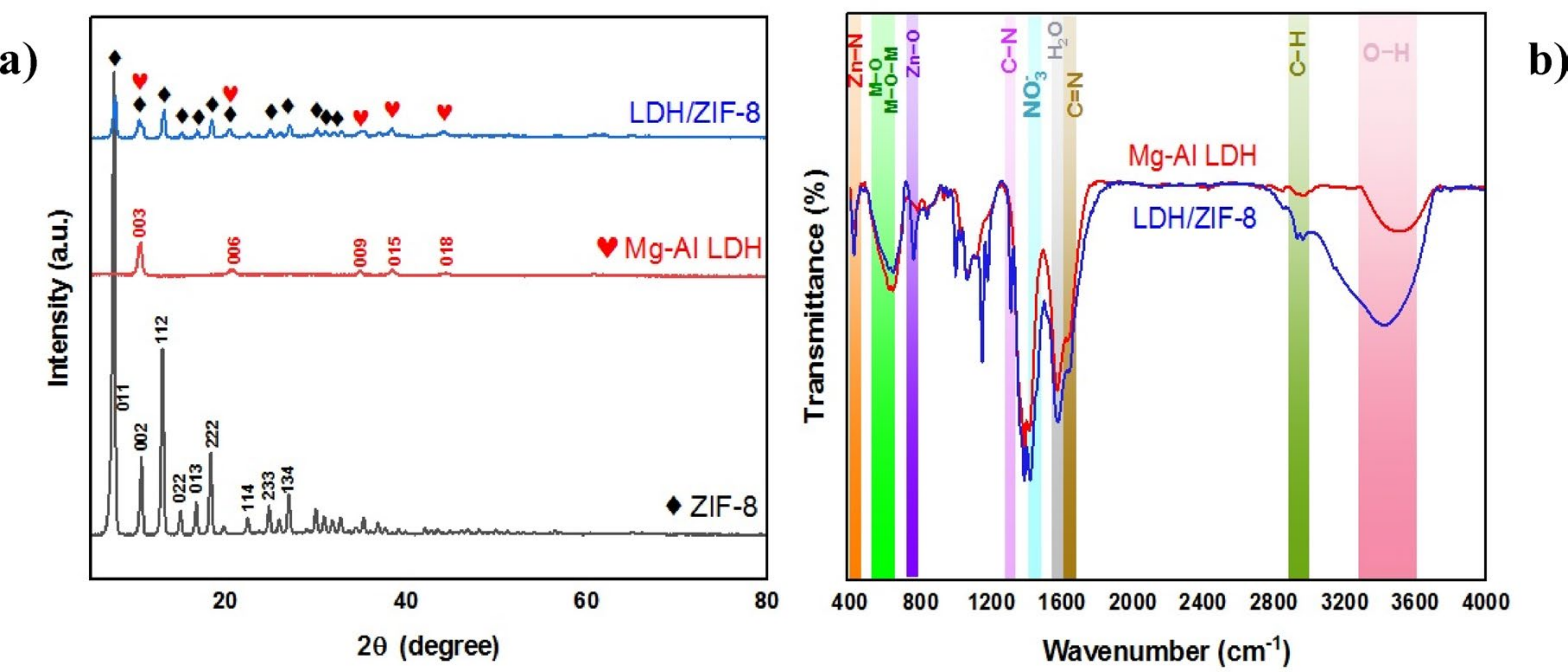
(**a**) XRD patterns and (**b**) FTIR spectra of different samples.

XRD was used to examine the structures of Mg-Al LDH, ZIF-8, and Mg-Al LDH/ZIF-8 (Fig. 1a). In XRD patterns of Mg-Al LDH, the characteristic peaks of crystal planes of (003), (006), (009), (015), (018), (110), and (113) are observed which correspond to JCPDS No.0191 − 14^37,38^. In addition, the diffraction pattern of ZIF-8 shows the crystal planes of (011), (002), (112), (022), (013), (222), (114), (233), and (134) correspond to the peaks of 7.4, 10.48, 12.8, 14.8, 16.56, 18, 22.2, 24.6, and 25.7° (JCPDS No. 1030-062−00)^39,40^. In XRD pattern of the LDH/ZIF-8 nanocomposite, the peaks of LDH and ZIF-8 can be observed due to the successful formation of nanocomposite.

Figure 1b shows the FTIR analysis of the Mg-Al LDH and Mg-Al LDH/ZIF-8. For LDH/ZIF-8, the absorption peaks at 1574 and 643 cm^− 1^ are attributed to the vibrations of interlayer water molecules and the lattice vibrations of M–O–M and M–O bonds, respectively. Additionally, the peak at 1416 cm^− 1^ is due to vibrations of nitrate ions (NO_3_^−^). The stretching vibrations of C = N and C − N bonds at 1641 and 1308 cm^− 1^ are due to imidazole rings, respectively. The vibrations of Zn − O and Zn − N bonds in ZIF-8 structure are observed at 759 and 420 cm^− 1^, respectively. Experimental results indicate that the nanocomposite was successfully synthesized and that the resulting structural characteristics align with the predicted specifications^39^.

**Fig. 2:**
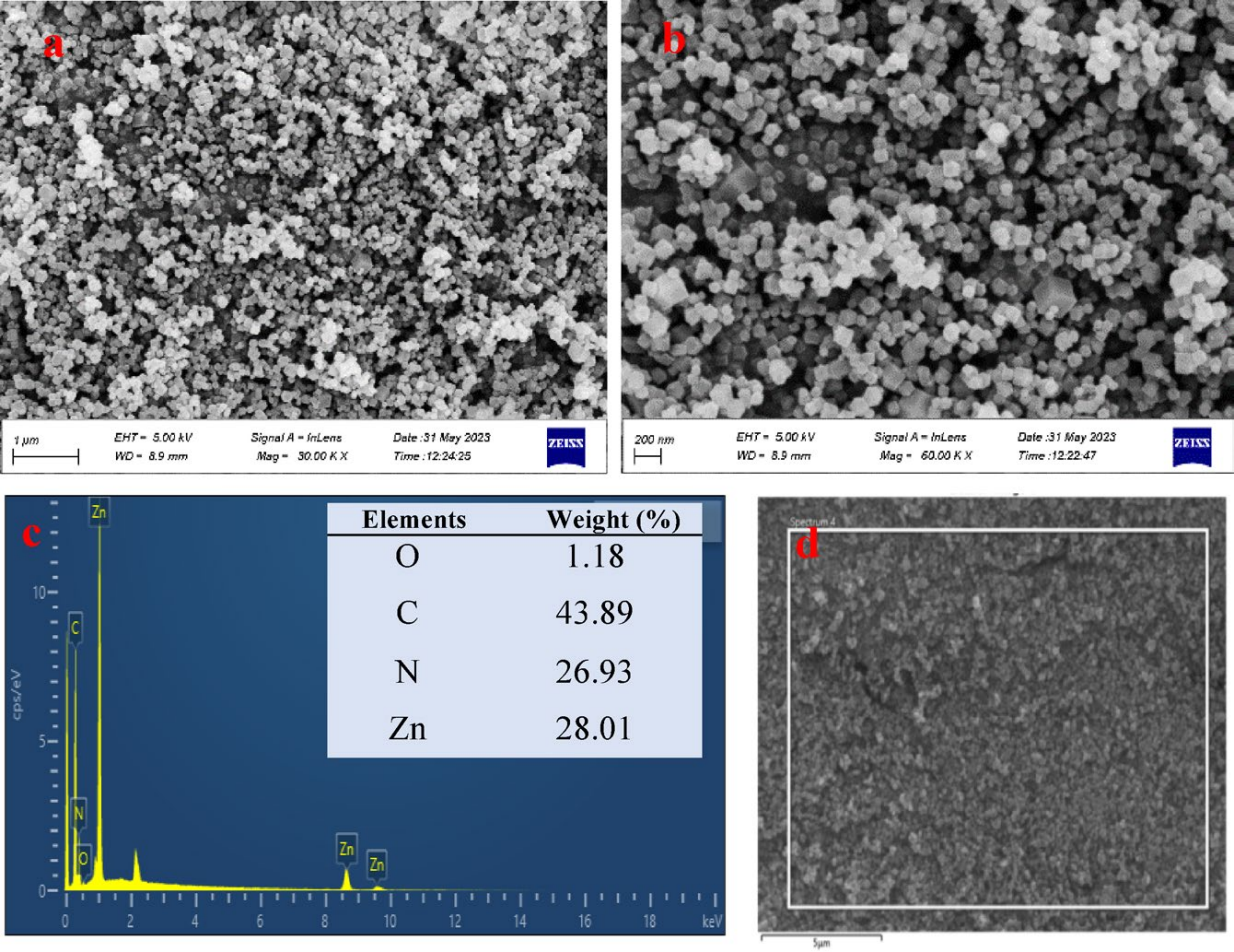
(**a**, **b**) FESEM images of ZIF-8, (**c**) EDS of (**d**) FESEM image of selected area (Inset: element percentage).

**Fig. 3:**
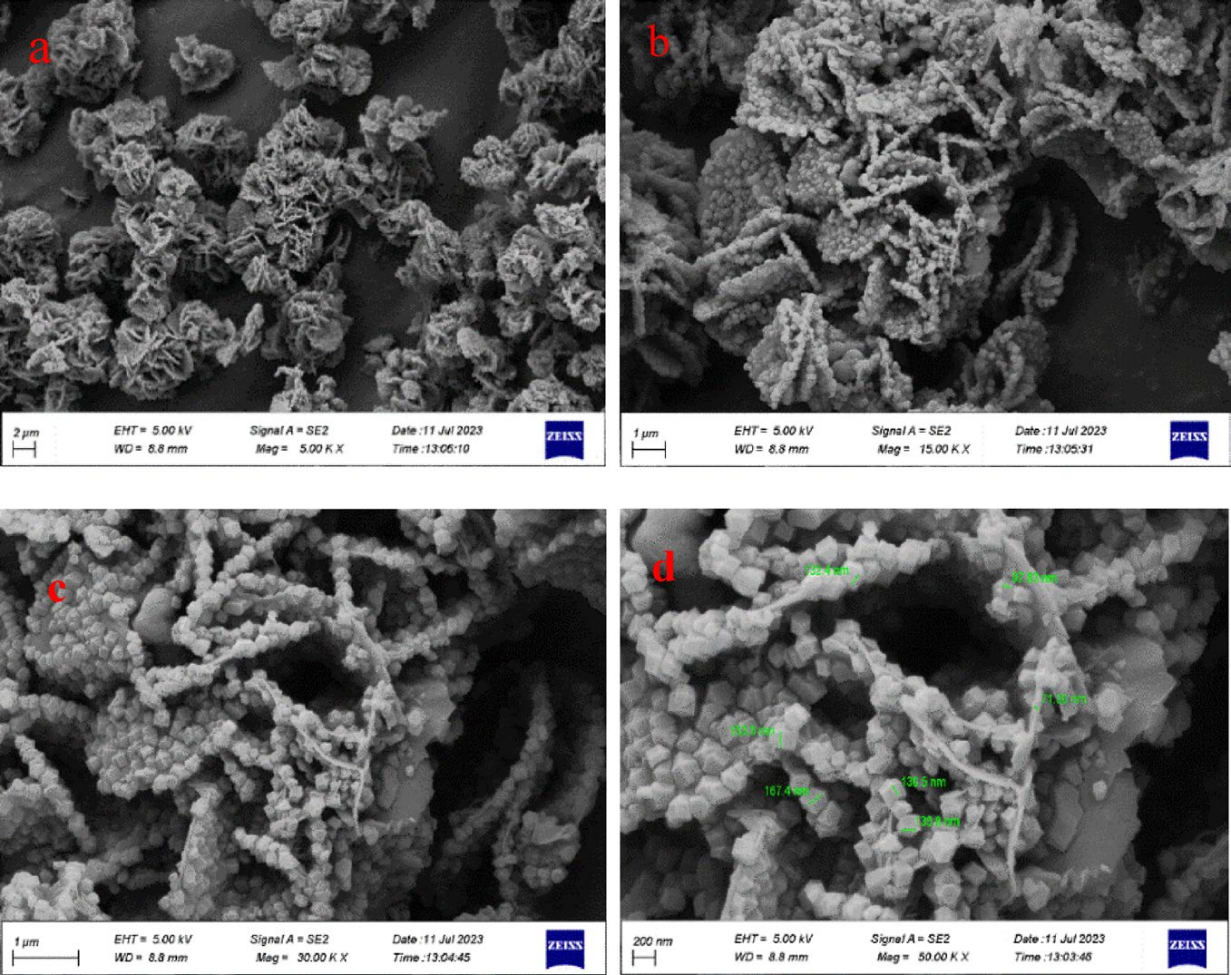
FESEM images of ZIF-8 (**a** and **b**) and Mg-Al LDH/ZIF-8 (c-f) at different magnifications.

FESEM images of ZIF-8 are presented in Fig. 2. The morphology of sample consists of polyhedral crystals with uniform size. Moreover, EDX spectrum of selected area in ZIF-8 shows the presence of K_α_ and L_α_ of Zn as well as the K_α_ lines of C, N and O elements which arises of 2-MIM and NO_3_^−^. Moreover,

Figure 3 shows FESEM images of LDH/ZIF-8 nanocomposite at different magnifications. The sheet-like structure of LDH and polyhedral structures of ZIF-8 are observed on the surface^41^. Their homogeneous distribution contributes to increased surface roughness and porosity, creating abundant active sites for adsorption and catalytic reactions. Such structural synergy between the thin LDH nanosheets and well-dispersed ZIF-8 crystals significantly improves the surface area and accessibility of reactive regions. This combination favors enhanced mass transport, higher pollutant interaction efficiency, and makes the nanocomposite an excellent candidate for environmental remediation.

**Fig. 4:**
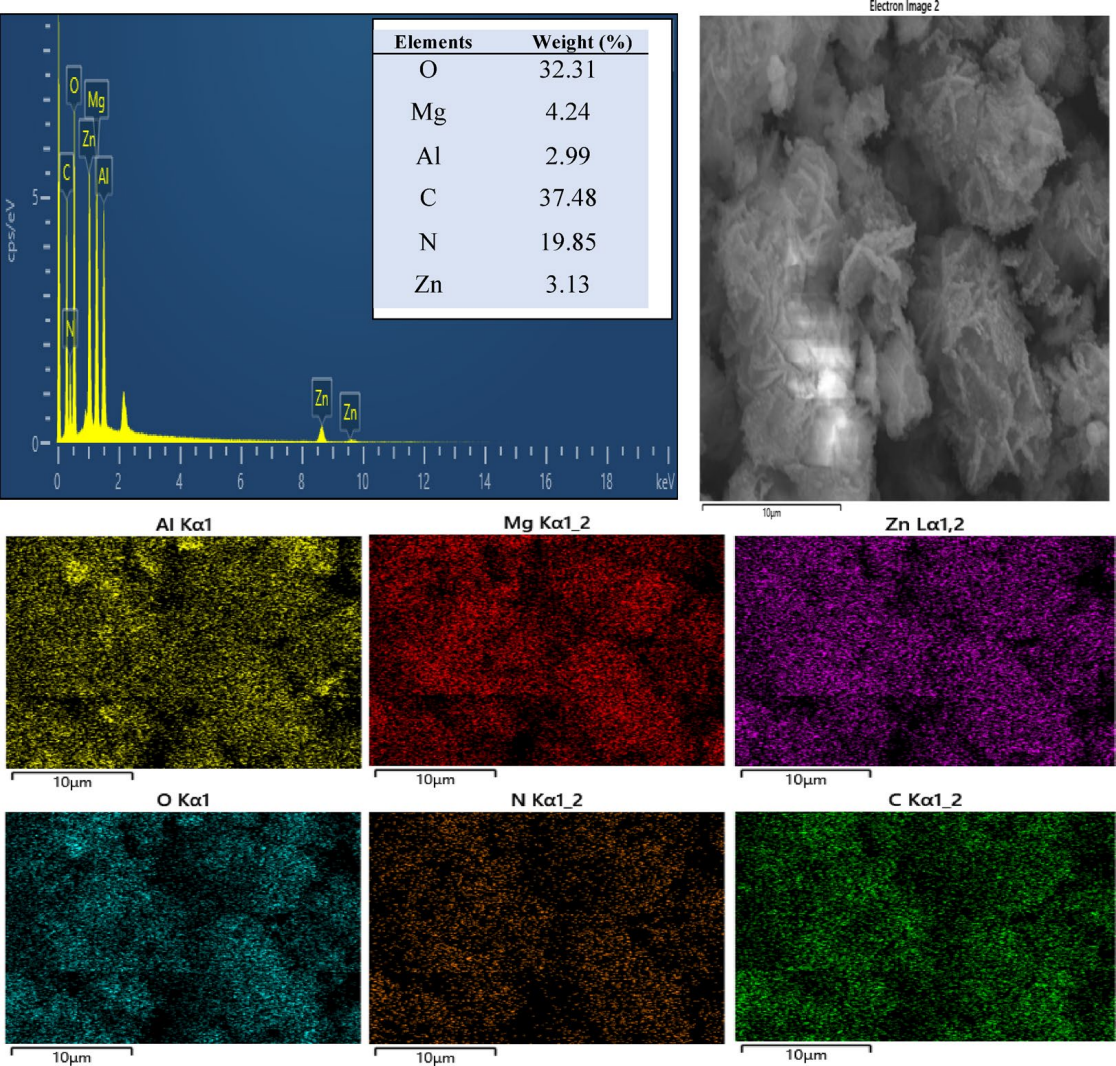
EDX spectrum and mapping analysis of selected area in LDH/ZIF-8. (Inset: element percentage).

Figure 4 shows the EDX analysis and elemental mapping images of Mg-Al LDH/ZIF-8 nanocomposite. The peaks of magnesium (Mg), aluminum (Al), oxygen (O), zinc (Zn), and nitrogen (N) are clearly detected, verifying the successful synthesis of Mg-Al LDH and ZIF-8 components (table inset). Elemental mapping reveals a uniform distribution of these elements across the composite surface, indicating strong interfacial contact and homogeneous hybridization among LDH nanosheets and ZIF-8 nanocrystals.

**Fig. 5:**
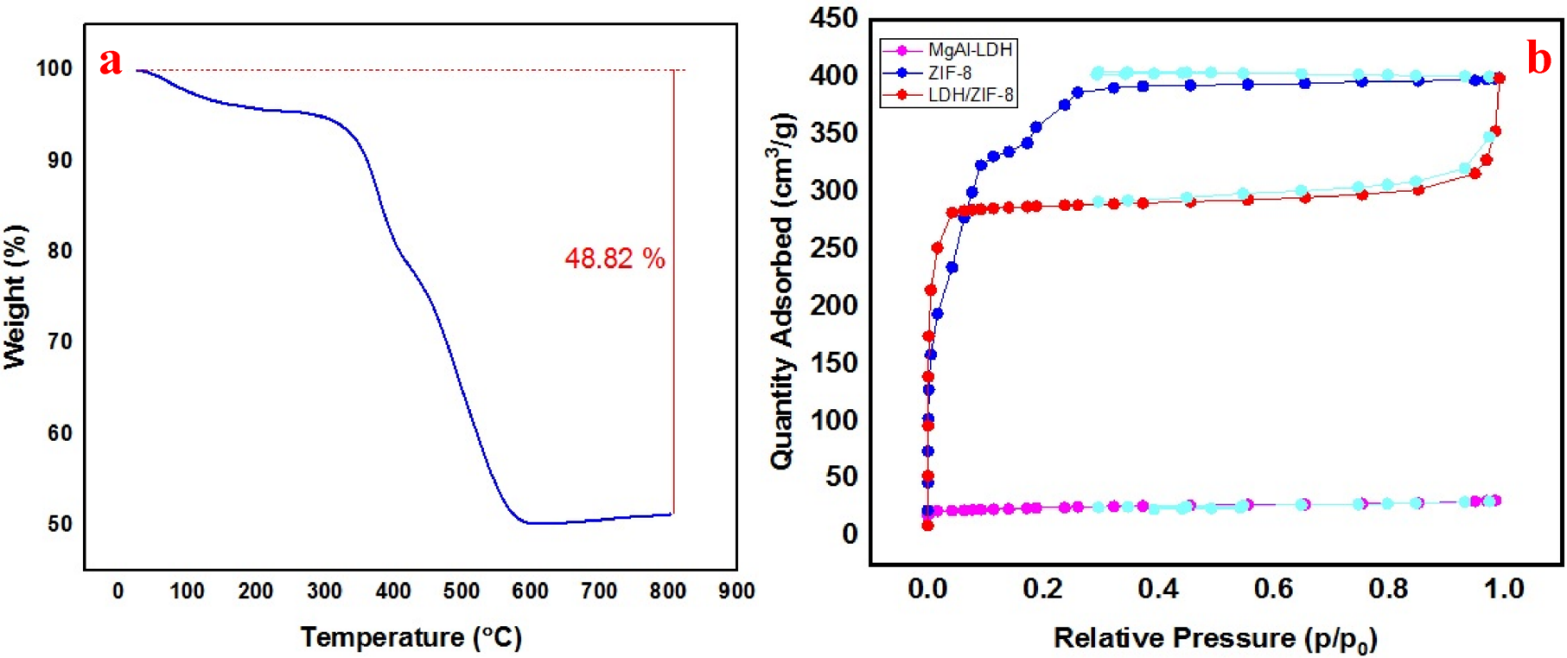
(**a**) TGA curve of LDH/ZIF-8. (**b**) N_2_ adsorption/desorption isotherms of Mg-Al LDH, ZIF-8 and Mg-Al LDH/ZIF-8.

Figure 5a shows the results of TGA of Mg-Al LDH/ZIF-8 performed under argon atmosphere. TGA curve displays that the sample undergoes multiple distinct stages of weight changes, each corresponding to specific decompositions or alterations in the chemical composition. The most significant feature of the TGA is the weight loss of approximately 5–10% occurring around 50–150 °C. In this step, surface-absorbed water and physical water evaporate due to the escape from the surface and interlayer structure of Mg-Al LDH. The weight loss in the range of 200–400 °C is attributed to removal of hydroxyl groups and decomposition of organic ligand in ZIF-8 structure. Moreover, a weight loss of about 30–40% in the temperature range of 400–600 °C are due to the decomposition of the ZIF-8 and other organic materials including solvent. At 600 °C and above, weight loss becomes minimal or constant, indicating the material reaches a stable state with no significant weight changes observed^42,43^. These findings reveal highlight the structural transitions of the nanocomposite.

Figure 5b depicts the isotherms of N₂ adsorption/desorption recorded at 77 K. For Mg-Al LDH, a type IV isotherm with H4 hysteresis is observed due to its mesoporous and layered structures. The Mg-Al LDH/ZIF-8 nanocomposite presents a combination of type I isotherm (micro-porous characteristics from ZIF) and type IV isotherm (mesoporous features from LDH), resulting in an enhanced specific surface area (Table 3). Data obtained from Brunauer–Emmett–Teller (BET) plot revealed that the nanocomposite possesses higher surface area and porosity than Mg–Al LDH. This improvement is primarily attributed to the integration of ZIF-8, which inherently features a highly porous architecture and large surface area.

**Table 3:** Textural information obtained from N_2_ adsorption/desorption isotherms.

Sample	BET (m^2^/g)	Average pore diameter (nm)	Total pore volume (cm^3^/g)
Mg-Al LDH	83.044	2.3235	0.04824
ZIF-8	1852.7	1.3517	0.6261
Mg-Al LDH/ZIF-8	868.3	2.0118	0.4367

### Model validation and statistical analysis

The validation of the models was conducted using ANOVA, as elaborated in Table 4. The Eq. (2) shows the effect of antibiotic concentration, Mg-Al LDH/ZIF-8 dosage, pH, and contact time on removal efficiency. All variables exhibit statistically significant effects. The validity of the second-order model is confirmed through the data in Table 4, with reference to Table 1.

**Table 4:** ANOVA outputs for TC elimination model.

Source	Sum of Squares	df		Mean Square	F-value	*p*-value	
Model	5573.66	11	506.70		51.08	< 0.0001	significant
A-Concentration	1046.76	1	1046.76		105.52	< 0.0001	
B-Time	853.23	1	853.23		86.01	< 0.0001	
C-Catalyst Dosage	1068.00	1	1068.00		107.66	< 0.0001	
D-PMS Dosage	470.82	1	470.82		47.46	< 0.0001	
E-pH	117.48	1	117.48		11.84	0.0026	
AC	172.27	1	172.27		17.37	0.0005	
CD	158.13	1	158.13		15.94	0.0007	
DE	160.66	1	160.66		16.20	0.0007	
C^2^	1116.50	1	1116.50		112.55	< 0.0001	
D^2^	339.43	1	339.43		34.22	< 0.0001	
E^2^	246.43	1	246.43		24.84	< 0.0001	
Residual	198.40	20	9.92				
Lack of Fit	134.87	15	8.99		0.7076	0.7246	not significant
Pure Error	63.53	5	12.71				
Cor Total	5772.06	31					

The model’s reliability is supported by the coefficient of determination (R²), with values of R² = 0.9656, adjusted R² = 0.9467, and predicted R² = 0.9142, indicating a high level of accuracy in the proposed equations.

2 $${\text{Y(\% RE) = - 144}}{\text{.738 + 0}}{\text{.259A + 2}}{\text{.385 B + 14}}{\text{.984 C + 292}}{\text{.321 D + 24}}{\text{.558 E - 0}}{\text{.088 AC + 11}}{\text{.644 CD - 23}}{\text{.472 DE - 0}}{\text{.680 C}}^{2} {\text{ - 414}}{\text{.26 D}}^{2} {\text{ - 1}}{\text{.280 E}}^{2} {\text{ }}$$

**Fig. 6:**
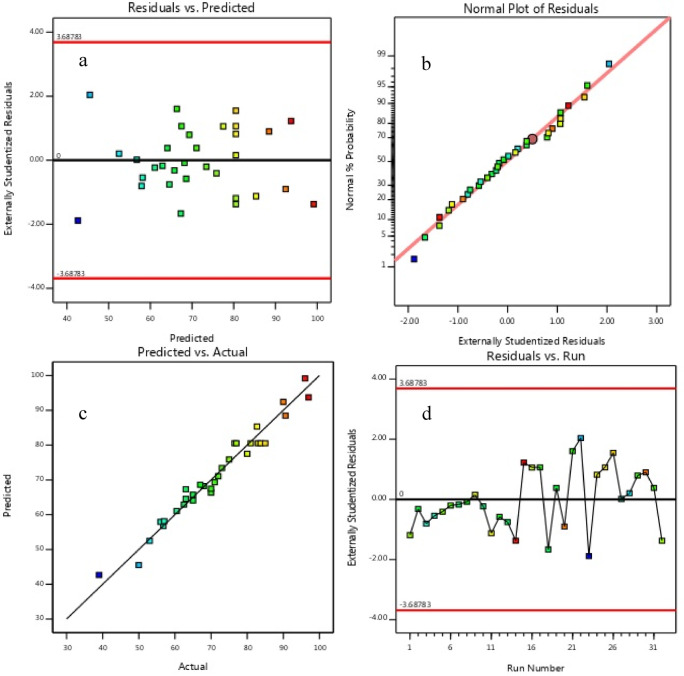
Plots of (**a**) residual versus predicted, (**b**) normal of residuals, (**c**) predicted versus real data, and (**d**) residual versus run number.

Model adequacy was further evaluated through the residual plots (Fig. 6) which show the variations between predicted and observed responses and are essential for evaluating model adequacy. In Fig. 6a, the residuals follow the red reference line, indicating minimal systematic error and confirming the model’s validity. Another approach involves plotting residuals against fitted data. In Fig. 6b, the random dispersion of residuals around the reference line demonstrates the accuracy of the obtained model. In Fig. 6c, a discernible pattern in the plot would indicate non-random errors. Also, no apparent pattern is observed in the specified model. In Fig. 6d, the predicted line is compared with both experimental and predicted values, clearly demonstrating the model’s high predictive accuracy. The data points exhibit strong agreement with the model’s predictions and are predominantly concentrated near the perfect prediction line.

###  Effect of parameters on TC removal

**Fig. 7:**
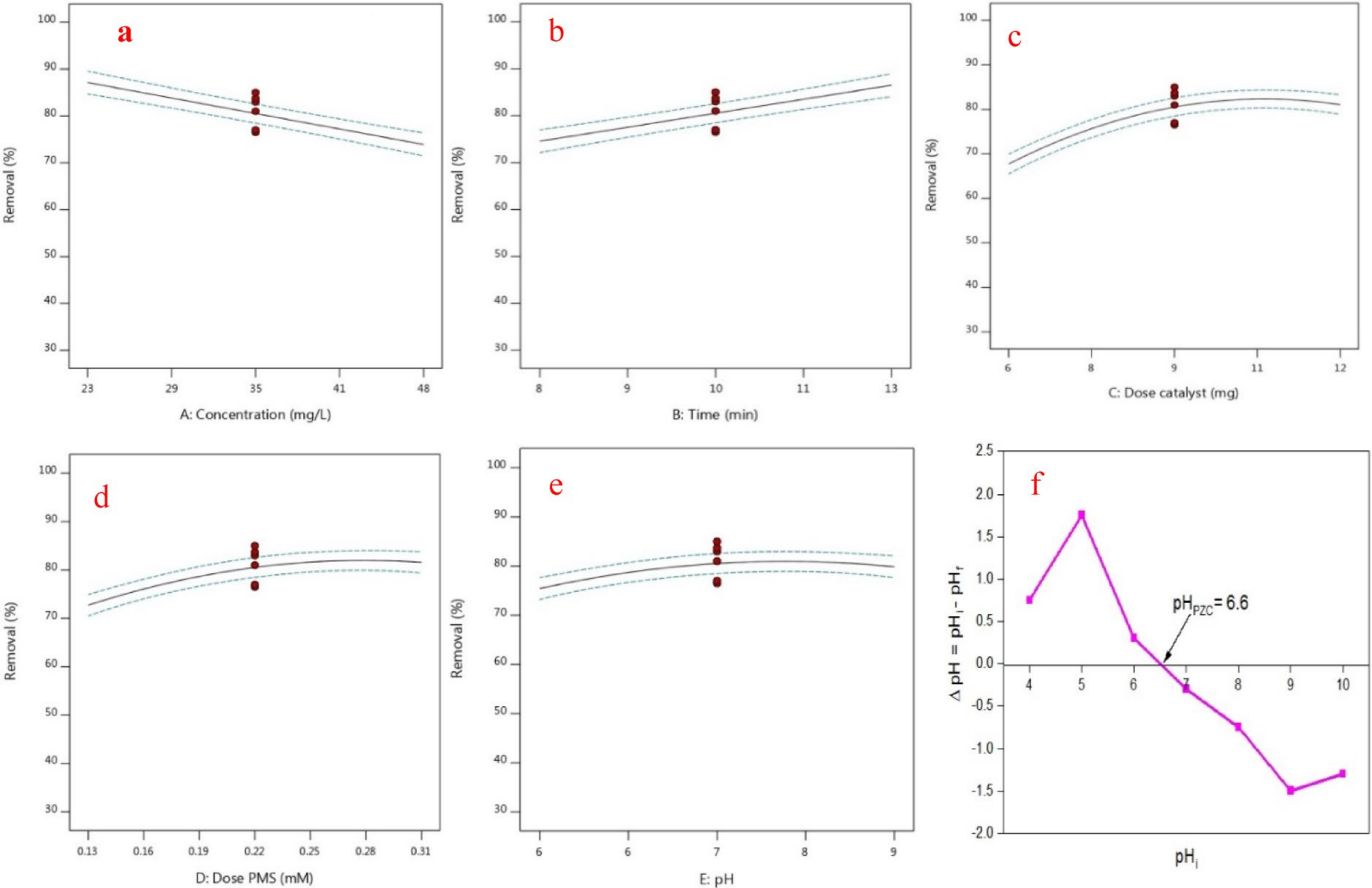
Effect of (**a**) initial concentration, (**b**) contact time, (**c**) catalyst dosage, (**d**) PMS dosage, (**e**) initial solution pH in TC removal and (**f**) determination of pH_pzc_ for LDH/ZIF-8.

According to Fig. 7a, the results from examining TC concentrations (ranging from 10 to 60 mg/L) reveal that this factor has a negative impact on the removal process. Since the removal efficiency depends on available sites, an increase TC concentration-while maintaing nanocomposit dosage and the number of active sites constant - leads to a higher number of pollutant molecules, consequently, results in a decrease in removal efficiency^44^.

In Fig. 7b, the effect of contact time on TC removal is displayed in the range of 5–15 min. The percentage of TC removal increases linearly with contact time. With increasing time of process, the likelihood of interaction of TC with the nanocomposite increases, thereby improving the removal efficiency.

Figure 7c demonstrates the impact of various nanocomposite dosages on TC removal within the range of 3–15 mg. The results indicate that increasing the nanocomposite dosage leads to a higher TC removal rate, this improvement might be due to the presence of vacant spaces in the nanocomposite under a constant pollutant concentration. With increasing the Mg-Al LDH/ZIF-8 dosage, more active sites are present on the sample surface. However, at lower nanocomposite dosages, the number of active sites may not be sufficient in comparison to the pollutant molecules which lead to reduced removal efficiency. At higher nanocomposite dosages, the efficiency stabilizes and eventually declines. This decrease is likely caused by the aggregation of nanocomposite particles, which block the available active sites and hinder the removal process^45^.

Figure 7d illustrates the impact of PMS dosages on TC removal within the range of 0.04–0.4 mM. Generally, PMS promotes the generation of free radicals, thereby enhancing the removal efficiency of the nanocomposite. However, when the PMS dosage exceeds the optimal level, the removal efficiency shows no substantial improvement. This can be attributed to the reaction between excessive PMS and SO_4_^−^ as well as OH^−^, leading to the formation of SO_5_^−^, which is a relatively weak oxidant. Consequently, this diminishes the TC removal performance^46^.

Figure 7e displays the effect of pH changes on TC removal. The pH changes were analyzed within the range of 4–10. TC has three dissociation constants (3.3, 7.68 and 9.96), exhibiting different forms under varying pH conditions^47^. Based on the figure, the removal efficiency initially increases with rising pH but subsequently declines due to the electrostatic interactions between the nanocomposite and TC. Figure 7f depicts the point Ofzero charge (PZC) value of the LDH/ZIF-8 nanocomposite, obtained through the solid addition method^48,49^, which elucidate the electrostatic interactions governing TC removal efficiency at different pH values.

### Optimization

The parameters of TC concentration, time, nanocomposite dose, PMS dose and solution pH were optimized using the RSM method to achieve maximum efficiency. The optimization was conducted based on Eq. 3, and the model predictions at the optimal conditions are summarized in Table 5. Furthermore, the proposed model shows good agreement with results obtained from experimental analysis.

**Table 5:** Anticipated and performed tests under optimal conditions.

	TC concentration (mg/L)	Contact time (min)	Nanocomposite dosage (mg)	PMS dosage (mM)	pH	Removal (%)
Predicted	25	12	9	0.28	7	100
Actual	25	12	9	0.28	7	97.5

### Effect of PMS

**Fig. 8:**
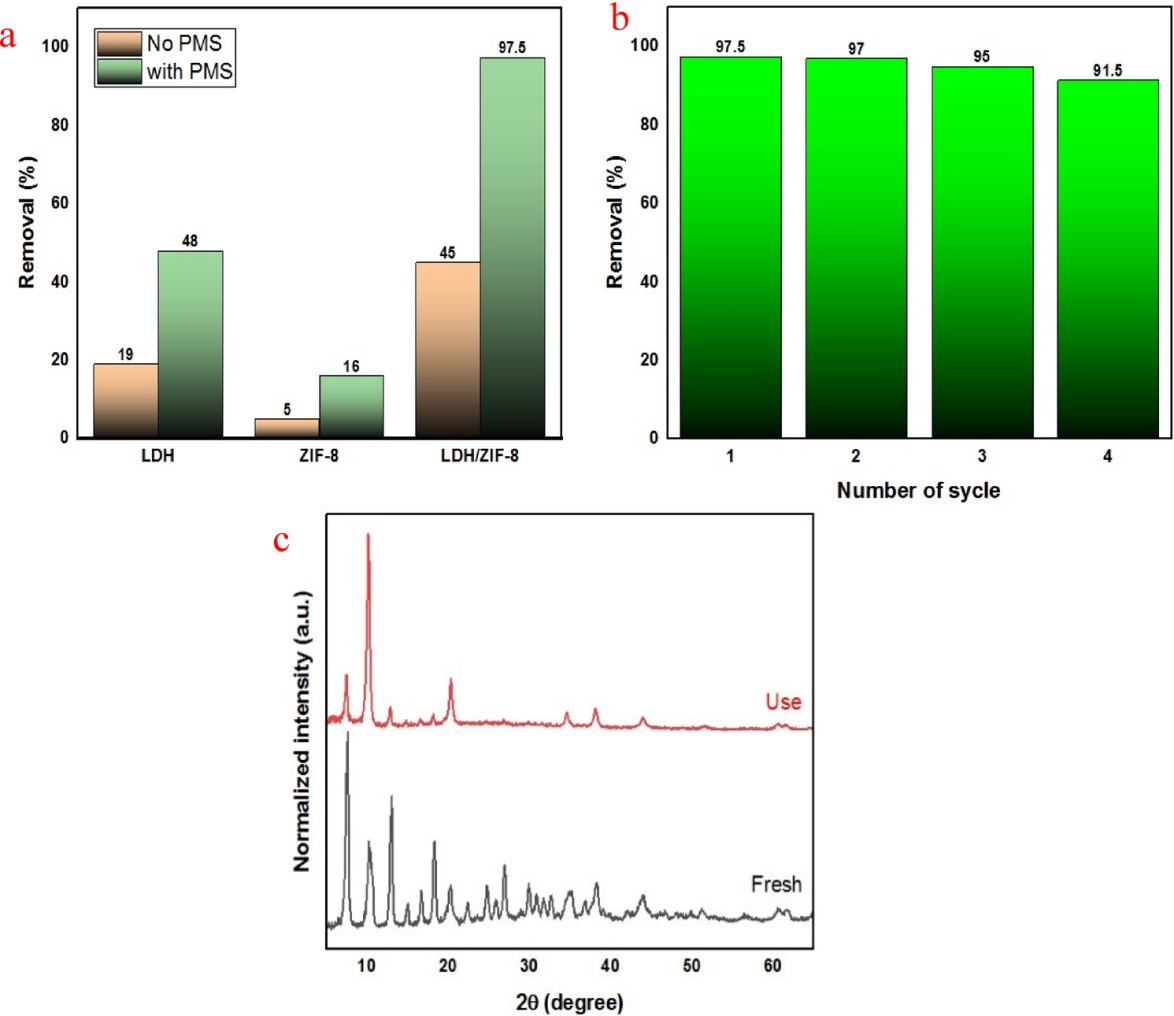
(**a**) Comparison of different materials for TC removal, (**b**) Recyclability tests of LDH/ZIF-8 and (**c**) XRD patterns of initial and recovered sample.

In Fig. 8a, the performance of LDH, ZIF-8, and LDH/ZIF-8 samples for TC removal was evaluated in the presence of PMS under parametrically optimized conditions. For LDH and ZIF-8 samples, the addition of PMS resulted in an increase in removal of 29 and 14%, respectively. Finally, it was found that LDH/ZIF-8 performed better than LDH and ZIF-8 samples, and the removal rate increased from 45 to 97.5% with the addition of PMS.

### Stability test

The stability and reusability of the Mg-Al LDH/ZIF-8 nanocomposite and its performance in removing TC were examined over four cycles. After each cycle, the sample was centrifuged, rinsed with ethanol, and dried at 70 °C. As depicted in Fig. 8b, the removal efficiency declined by about 6% after four successive cycles, likely due to the partial loss of Mg-Al LDH/ZIF-8 during the removal process or the washing stage. The structural integrity of the LDH/ZIF-8 nanocomposite was studied via XRD technique (Fig. 8c). Comparison of the peak positions in the diffraction patterns shows that the intensity of some peaks has decreased but the positions of some peaks have been maintained. These findings suggest that the LDH/ZIF-8 is an stable catalyst for the removal of TC from aqueous solutions.

### Kinetic study

The activity of LDH/ZIF-8 nanocomposite in the TC removal process was examined through two models: pseudo-second-order and pseudo-first-order kinetic equations outlined in Eqs. 3 and 4, respectively. The variables q_e_ and q_t_ (mg g^− 1^) represent the catalytic capacities at equilibrium and at a given time, t, while K_1_ (min^− 1^) and K_2_ (g mg^− 1^ min^− 1^) are the corresponding rate constants.

3 $$\:{log}\left({q}_{e}-{q}_{t}\right)={log}{q}_{e}-\frac{{K}_{1}}{2.303}t\:$$

4 $$\:\frac{{dq}_{t}}{dt}={K}_{2}{\left({q}_{e}-{q}_{t}\right)}^{2}\:$$

As shown in Table 6, for TC removal, the pseudo-second-order model demonstrated superior correlation with a higher R^2^ value, underscoring its relevance in describing the catalytic mechanism.

**Table 6:** Numerical results for kinetic models.

C_0_ (mg L^− 1^)	q_e, exp_ (mg g^− 1^)	Pseudo-first order					Pseudo-second order			
		q_e, cal_ (mg g^− 1^)	K_1_ (min^− 1^)	*R* ^2^		q_e, cal_ (mg g^− 1^)	K_2_ (mg g^− 1^ min^− 1^)		*R* ^2^	
10	54.81	21.13	0.235	0.9611		59.17	0.0163	0.9947		
20	104.11	41.21	0.227	0.9622		113.64	0.0082	0.9948		
30	147.83	61.66	0.228	0.9595		158.73	0.0056	0.9948		
40	185.98	82.22	0.227	0.9622		200	0.0042	0.9908		
50	218.44	74.99	0.227	0.9418		238.10	0.0031	0.9833		
60	248.59	123.03	0.228	0.9595		270.27	0.0026	0.9863		

### Isotherm study

The interaction of the LDH/ZIF-8 with TC was investigated through the Langmuir, Freundlich, and Temkin models. The Langmuir model is founded on the assumptions including (1) active sites on homogeneous surface possess uniform energy and (2) there are no interaction between molecules adsorbed as monolayer. This model is mathematically expressed as:

5 $$\:\frac{{C}_{e}}{{q}_{e}}=\frac{1}{{q}_{\mathrm{m}\mathrm{a}\mathrm{x}}{K}_{L}}+\frac{{C}_{e}}{{q}_{\mathrm{m}\mathrm{a}\mathrm{x}}}\:$$

Where q_e_ and q_max_ (mg g^− 1^) denote the equilibrium coverage and the maximum capacity of the catalytic surface, respectively, C_e_ (mg L^− 1^) represents the equilibrium concentration of TC in solution, and K_L_ (L mg^− 1^) is the equilibrium constant that characterizes the affinity between the catalytic surface and the target compound. This equation provides a quantitative description of the interaction dynamics, offering insights into the efficiency and mechanistic aspects of the catalytic process.

In Freundlich model, non-ideal and multilayer interactions on a heterogeneous surface are considered. It represented as:

6 $$\:{ln}\left({q}_{e}\right)=\frac{1}{n}{ln}\left({c}_{e}\right)+{ln}\left({K}_{F}\right)\:$$

Hence n and K_F_ (L g^− 1^) are Freundlich constants.

Also, Temkin isotherm model can be written using mathematical equation as below:

7 $$\:{q}_{e}={B}_{T}Ln{K}_{T}+{B}_{T}{ln}{C}_{e}\:$$

Hence, B_T_ and K_T_ are the Temkin equation constant; T is temperature (K), and R is general gas constant (8.314 J mol^− 1^ K^− 1^).

As shown in Table 8, the Langmuir model exhibit the highest correlation coefficient (R^2^ = 0.9923), indicating its strong alignment with the removal process. This high correlation indicates that the interaction follows through the adsorption of antibiotic monolayer on the LDH/ZIF-8 surface, making the Langmuir model more suitable for further investigating the functional mechanism of the system.

Table 7 shows the effect of PMS concentration in the range of 10 to 60 mg/L. The highest efficiency was calculated to be 322.58 mg/g, based on the Langmuir constant, demonstrating the high capability of nanocomposite in facilitating the removal process. Table 8 shows the comparison of different nanocomposites for PMS activation for TC removal. As can be seen, the LDH/ZIF-8 nanocomposite has the highest TC removal and shows a promising application perspective.

**Table 7:** Parameters obtained from fitting equilibrium data with different isotherms.

Isotherm model	Parameters	Amounts
Langmuir	q_m_ (mg g^− 1^)	322.58
	K_L_ (L mg^− 1^)	0.146
	R^2^	0.9923
Freundlich	n	1.87
	K_F_ (mg/g) (L/mg)^1/n^	52.196
	R^2^	0.9897
Temkin	B_T_	68.194
	K_T_ (L mg^− 1^)	1.568
	R^2^	0.9866

**Table 8:** Comparison of performance of different catalysts for TC removal.

Nanocomposite	Removal (%)	Ref
Fe-NPC	90	^50^
Zero-valent iron (Fe^0^)	88.5	^11^
FONC@PAC	86.9	^51^
Fe–Mn	90.7	^52^
Co/Cu co-substituted ferrite-carbon	90.06	^53^
CuBi_2_O_4_/BiOBr	90.3	^54^
Mg-Al LDH/ZIF-8	97.5	This work

### Catalytic mechanism

In this study, the activation of PMS was examined by Mg-Al LDH/ZIF-8 nanocomposite for the removal of TC under optimal conditions. Upon activation of PMS by the nanocomposite, multiple ROS were generated, including sulfate radicals${\mathrm{(SO}}_{{\mathrm{4}}} \bullet ^{ - } {\mathrm{)}}$, hydroxyl radicals$\left( { \bullet {\mathrm{OH}}} \right)$, and superoxide radicals $\left( {{\mathrm{O}}_{2} \bullet ^{ - } } \right)$. To identify the dominant ROS responsible for TC degradation, selective scavengers were introduced:

- Methanol (MeOH) quenches both ${\mathrm{(SO}}_{{\mathrm{4}}} \bullet ^{ - } {\mathrm{)}}$ and $\left( { \bullet {\mathrm{OH}}} \right)$.
- Diammonium oxalate monohydrate (DAOM) selectively quenches ${\mathrm{(SO}}_{{\mathrm{4}}} \bullet ^{ - } {\mathrm{)}}$.
- Benzoquinone (BQ) selectively quenches $\left( {{\mathrm{O}}_{2} \bullet ^{ - } } \right)$.

The proposed reactions are as follows:

$${\mathrm{HSO}}_{5} ^{ - } + {\mathrm{Mg}}^{{2^{ + } }} \to {\mathrm{SO}}_{4} \bullet ^{ - } + {\mathrm{OH}}^{ - } + {\mathrm{Mg}}^{{3^{ + } }}$$

$${\mathrm{HSO}}_{5} ^{ - } + {\mathrm{Al}}^{{3^{ + } }} \to {\mathrm{SO}}_{4} \bullet ^{ - } + {\mathrm{OH}}^{ - } + {\mathrm{Al}}^{{4^{ + } }}$$

$${\mathrm{SO}}_{4} \bullet ^{ - } + {\mathrm{H}}_{{\mathrm{2}}} {\mathrm{O}} \to \bullet {\mathrm{OH}} + {\mathrm{HSO}}_{4} ^{ - }$$

$${\mathrm{O}}_{{\mathrm{2}}} + {\mathrm{e}}^{ - } \to {\mathrm{O}}_{{\mathrm{2}}} \bullet ^{ - }$$

$${\mathrm{TC}} + {\mathrm{SO}}_{4} \bullet ^{ - } / \bullet {\mathrm{OH}}/{\mathrm{O}}_{{\mathrm{2}}} \bullet ^{ - } \to {\mathrm{Intermediates}} \to {\mathrm{CO}}_{{\mathrm{2}}} + {\mathrm{H}}_{{\mathrm{2}}} {\mathrm{O}} + {\text{mineralized residues}}{\mathrm{.}}$$

**Fig. 9:**
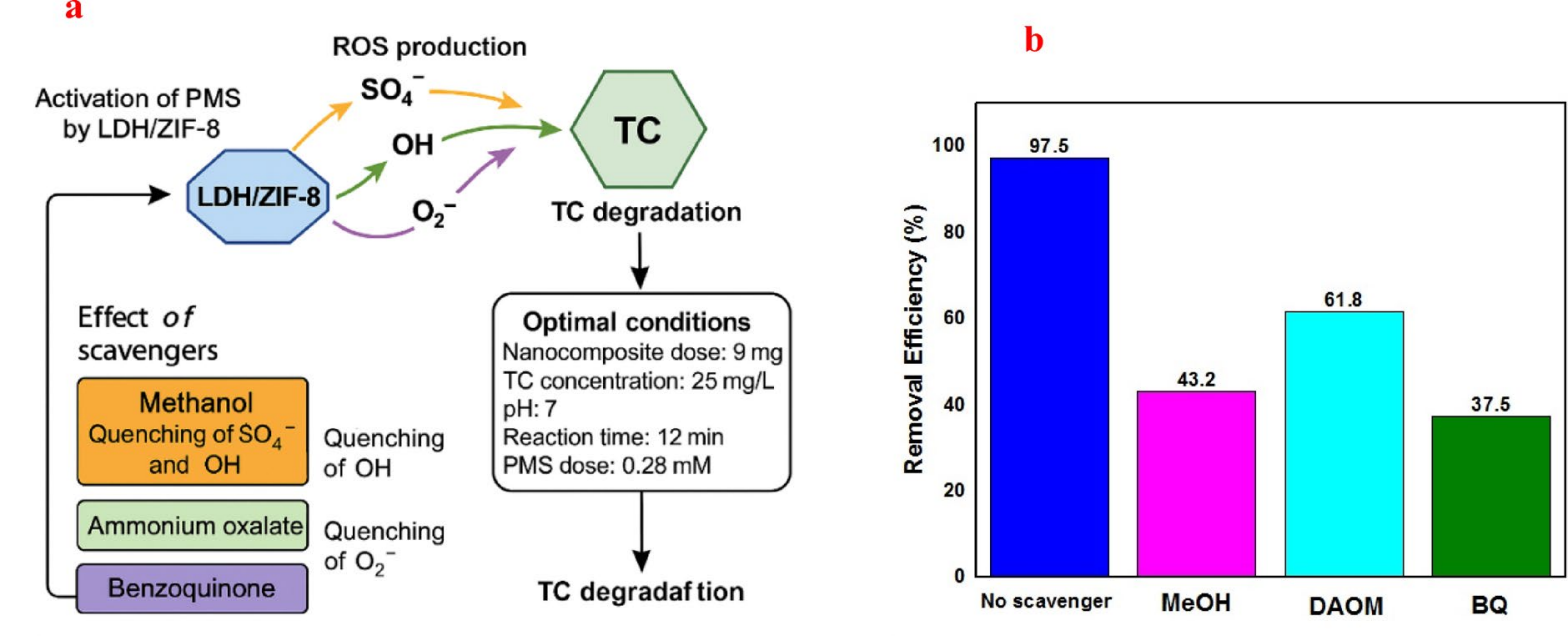
(**a**) Proposedmechanism for PMS activation and ROSProduction in Mg-Al LDH/ZIF-8 system. (**b**) Effect of different scavengers on TC removal .

Figure 9a shows the proposed mechanism for PMS activation and ROS generation. According to Fig. 9b the significant drop in TC removal upon MeOH addition confirms the dominant role of SO₄•⁻ and •OH radicals in the degradation process. The moderate decrease with DAOM indicates that SO₄•⁻ is a major contributor, while the relatively lower impact of BQ suggests that O₂•⁻ has a secondary role. Therefore, the degradation of TC is primarily driven by sulfate and hydroxyl radicals generated via PMS activation by LDH/ZIF-8, with superoxide radicals providing a complementary oxidative pathway.

##  Conclusions

In this study, the Mg-Al LDH/ZIF-8 was prepared and used to remove TC antibiotic from aqueous solution. Moreover, the role of nanocomposite as an activating agent for PMS was extensively studied to enhance the removal efficiency. The optimization of initial concentration of the contaminant, time, nanocomposite dosage, PMS dosage and pH were investigated and modeled using RSM. The highest removal of TC (97.5%) were obtained at optimized conditions of pH = 7, nanocomposite dosage = 9 mg, PMS dosage = 0.28 mM, initial concentration = 25 mg/L, and contact time = 12 min. The Mg Al LDH/ZIF-8 nanocomposite has higher performance than the LDH and ZIF-8 samples in the presence of PMS. According to kinetic model, the removal of TC is primarily driven by chemical interactions and molecular processes between the LDH/ZIF-8 nanocomposite and TC molecules. Isotherm studies using different models were also performed to further evaluate the removal of antibiotic and provide robust evidence of the interaction between PMS, nanocomposite and TC molecules. To assess the stability and reusability of the Mg Al LDH/ZIF-8 sample in solution containing PMS, its performance was examined over four consecutive cycles. The removal efficiency experienced only a slight reduction of approximately 6% after four cycles, demonstrating the remarkable durability nanocomposite. This minimal decline was attributed to minor losses of nanocomposite components during the regeneration and washing stages. These findings suggest that PMS played a crucial role in minimizing structural degradation, thereby maintaining the effectiveness of the nanocomposite throughout repeated use.

## Data Availability

The datasets used and/or analyzed during the current study are available from the corresponding author on reasonable request.
